# A missense mutation in the highly conserved TNF-like domain of Ectodysplasin A is the candidate causative variant for X-linked hypohidrotic ectodermal dysplasia in Limousin cattle: Clinical, histological, and molecular analyses

**DOI:** 10.1371/journal.pone.0291411

**Published:** 2024-01-22

**Authors:** Frederik Krull, Martina Bleyer, Jana Schäfer, Bertram Brenig

**Affiliations:** 1 Institute of Veterinary Medicine, Georg-August University Goettingen, Goettingen, Germany; 2 German Primate Center, Pathology Unit, Goettingen, Germany; Visayas State University, PHILIPPINES

## Abstract

Ectodysplasin A related hypohidrotic ectodermal dysplasia (XLHED) is a well-studied fetal developmental disorder in mammals that mainly affects ectodermal structures. It has been identified in a variety of species, including mice, rats, dogs, cattle, and humans. Here, we report the clinical, histological, and molecular biological analyses of a case of XLHED in Limousin cattle. An affected Limousin calf showed pathognomonic signs of ectodermal dysplasia, i.e. sparse hair and characteristic dental aplasia. Histopathologic comparison of hairy and glabrous skin and computed tomography of the mandible confirmed the phenotypic diagnosis. In addition, a keratoconjunctivitis sicca was noted in one eye, which was also confirmed histopathologically. To identify the causative variant, we resequenced the bovine X-chromosomal ectodysplasin A gene (*EDA*) of the affected calf and compared the sequences to the bovine reference genome. A single missense variant (rs439722471) at position X:g.80411716T>C (ARS-UCD1.3) was identified. The variant resulted in an amino acid substitution from glutamic acid to glycine within the highly conserved TNF-like domain. To rule out the possibility that the variant was relatively common in the cattle population we genotyped 2,016 individuals including 40% Limousin cattle by fluorescence resonance energy transfer analysis. We also tested 5,116 multibreed samples from Run9 of the 1000 Bull Genomes Project for the said variant. The variant was not detected in any of the cattle tested, confirming the assumption that it was the causative variant. This is the first report of Ectodysplasin A related hypohidrotic ectodermal dysplasia in Limousin cattle and the description of a novel causal variant in cattle.

## Introduction

Hypohidrotic ectodermal dysplasia (HED) or “Christ-Siemens-Touraine syndrome” is an embryonic developmental disorder of the skin structures [1]. These developmental disorders are usually monogenic and recessively inherited and are most commonly caused by alterations in the ectodysplasin A pathway [2–7]. This applies to the skin and its appendages, i.e. hair and nails, as well as all superficial glands that may be missing or maldeveloped [8, 9]. Affected animals have a sparse, usually lightened coat, malformed or missing teeth, and cannot regulate their body temperature by sweating (hypohidrosis) [1, 10]. Calves are characterized by bald patches around the eyes and muzzle and lack teeth with the exception of one malformed caudal molar [11–15]. Mammalian skin is of mesodermal and ectodermal origin and consists of three layers, with the middle layer, the corium, having the most malformed structures. No other syndromic malformations have been described in calves and the animals show normal daily gains. When the causative mutation is located on the X chromosome, the disorder is termed X-linked hypohidrotic ectodermal dysplasia (XLHED) and is much more commonly observed in male individuals [6]. HED-related malformations do not result in abortion but often cause premature death from inefficient thermoregulation or pneumonia due to decreased pulmonary clearance because the appropriate glands are absent. HED may therefore be considered an indirect lethal factor in cattle [15, 16].

To date, there have been 11 reports of HED in cattle, i.e. Red or Black Holstein and various crosses of Red Angus, Charolais, Simmental, Japanese Black or British-blue [3–9, 11–17]. Penetrance of HED appears to be high and the phenotype is easily observed. All reports to date are due to single genetic variants that do not appear to be common in the rest of the population.

In addition to cattle, HED has been described in humans, dogs, rats, and various strains of tabby mice [2, 18–20]. Milder forms of HED have been observed in females [21]. In dogs, HED has been described in various breeds, such as German Shepherds, Dachshunds, Poodles, and mixed breeds [22–25]. The prevalence of the disease has not been estimated in animals, but in humans, the disease occurred in 1 of 17,000 children [26].

The bovine Ectodysplasin A gene *EDA* is located at position X:g.80,405,885–80,803,322 (ARS-UCD1.3), spans approximately 397kb and consists of eight exons. The encoded 391 amino acid harboring Ectodysplasin A protein contains a collagen-like domain that mediates homotrimerization and a tumor necrosis factor (TNF)-like domain for receptor interaction. Ectodysplasin A is a paracrine cytokine, relevant to cell-cell communication, particularly in tissues of mesodermal and ectodermal origin [2, 27]. The protein is either located on the cell surface or secreted by furin cleavage [28]. Because of an alternative splicing process, there are two isoforms of Ectodysplasin A A1 and A2, which differ only in residues 308–309. These two residues are located within the TNF-like domain and therefore affect the receptor specificity of the two isoforms [27]. This pattern is highly conserved and is found in various mammals, such as humans and mice [29, 30].

Interestingly, the two isoforms activate different cellular receptors. Isoform A1 activates the Ectodysplasin A receptor (EDAR), whereas isoform A2 has no effect on this receptor, but activates only the X-linked ectodysplasin A receptor (XEDAR) [31]. Only isoform A1 appears to be involved in the pathway relevant to HED, as mutations of EDAR also lead to HED, but with similar heritability in both sexes, as EDAR is an autosomal gene on chromosome 11 [32]. An intracellular downstream mediating receptor for EDAR, the EDAR-associated death domain (EDARADD), also causes autosomal HED when disrupted [33]. Isoform A2 selectively activates the X-linked EDA-receptor (XEDAR) whose biological functions are still poorly understood but which also appears to be important in skin development and immune responses by activating various intercellular signaling pathways [34, 35]. XEDAR mutations, cause only a mild form, if any, of HED [36]. XEDAR knock-out mice are indistinguishable from healthy mice [30]. Other isoforms of EDA have been shown to be expressed but do not appear to have biological functions [19]. A similar form of HED, which is affected by the Wnt pathway, has been described in humans by mutations in the *WNT10A* gene [37]. As ectodysplasin A, Wnt is important for cell-cell communication at the embryonic stage. Eleven reports of HED in cattle are collected in OMIA, with currently only one report of HED due to mutations in EDAR [32, 38]. The remaining ten reports relate to XLHED due to EDA mutations, four of which describe small indels [5, 9, 11, 14]. Another six studies describe structural variations [6, 12, 13, 15–17].

To date, XLHED has not been reported in Limousin cattle. In the present study, we describe the genetic clarification of XLHED in an affected male calf from a family of purebred Limousin cattle.

## Material and methods

### Ethical statement

Samples were provided by a Limousin cattle breeder. Samples were taken exclusively by local veterinarians. The analysis of samples was approved by the Lower Saxony State Office for Consumer Protection and Food Safety (33.19-42502-05-17A196) according to §8a Abs. 1 Nr. 2 of the German Animal Protection Law.

### Sample collection and description

DNA of EDTA blood samples of the calf (46), its mother (44), its father (47), mothers`mother (43), and a healthy daughter of this grandmother (45) was extracted using MagNa Pure LC DNA Isolation Kit I (Roche Diagnostics, Mannheim, Germany). DNA of further unrelated Limousin cattle (n = 814) and Holstein cattle (n = 1,199) included in the study was obtained from our DNA depository. The affected calf was slaughtered at six months of age due to overall poor performance and skin samples of haired and bald areas from the forehead and an assumed blind eye were taken and fixed in 10% formalin and sent, processed routinely, and embedded in paraffin wax. Subsequently, 4μm sections were mounted on glass slides and stained with hematoxylin and eosin (H&E). The lower jaw was sent without fixation and computer tomographic images were taken on a Siemens SOMATOM Spirit 31164 system with syngo CT 2006C2 software. No clinical investigation of the animal by a veterinarian was conducted before slaughtering.

### PCR primer design and Sanger sequencing

PCR and Sanger sequencing were conducted in eight individuals containing the five Limousin family members (43–47) and three non-Limousin controls. PCR primers and reaction conditions are shown in Table 1. Primers were designed for each of the eight exons of the bovine EDA gene and transcript (NCBI NC_037357, NM_001081743.2, ARS-UCD1.3) using the PrimerQuest web tool (http://eu.idtdna.com/PrimerQuest) [39, 40]. Primers were synthesized by Sigma-Aldrich Chemie GmbH (Taufkirchen, Germany). PCR was optimized and performed using 30 cycles in a total volume of 20 μL, including 20 ng DNA, 10 μmol forward and reverse primer each, 1 × PCR reaction buffer (including 20 mM MgCl_2_), 100 μmol dNTPs and 0.6 U FastStart Taq DNA Polymerase (5 U/μl; Sigma-Aldrich Chemie GmbH, Taufkirchen, Germany) on a Biometra T gradient thermal cycler (Biometra, Göttingen, Germany). For exons 1, 3, and 4 a volume of 4 μL water was substituted with Q-solution (Qiagen, Hilden, Germany).

**Table 1:** PCR primers and probes.

Exon	Primer Name	Sequence (5`->3`)	Amplicon Size	Substitute	T_a_ (°C)
1	ED1_Exon1.1_for	GAAGGGCTGAGGCAGAC	252 bp	4μL Q-Solution	60
1	ED1_Exon1.1_rev	CAGTTCTAGGTAGCAGCACAA			
1	ED1_Exon1.2_for	TGGGTTTCTTTGGCCTCTC	310 bp	4μL Q-Solution	60
1	ED1_Exon1.2_rev	CTGCGCACATGGTGAGG			
2	ED1_Exon2_for	GTTGTTAGATGCCTTGCCAATAA	328 bp		60
2	ED1_Exon2_rev	CCTGGAAGCTATAGTACTCAAGAAG			
3	ED1_Exon3_for	CCAAGTTCCTTGAGGGTCATTA	506 bp	4μL Q-Solution	60
3	ED1_Exon3_rev	TCACCTGCTCCTGTTCTACTA			
4	ED1_Exon4.1_for	TTGACTGGGTCAACCTTTAACT	286 bp	4μL Q-Solution	57
4	ED1_Exon4.1_rev	TAGGTAGGTTAGGCTGGGAAA			
5	ED1_Exon5_for	GCTGCCTAGATGAAGAGGAAAG	183 bp		60
5	ED1_Exon5_rev	CTTAGCAGGGAGCAAACTCAA			
6	ED1_Exon6_for	AATGAGGCTCAGAGGCATTAC	351 bp		60
6	ED1_Exon6_rev	GGAACTAGGCTGGGTGATTATT			
7	ED1_Exon7_for	CCCAGATGATTCTGACATGTACT	234 bp		60
7	ED1_Exon7_rev	CAAAGGATCTGCATTCTGGATATAAG			
8	ED1_Exon8_for	ATGAGTGGGTCCTGTCTACT	545 bp		60
8	ED1_Exon8_rev	CCTGTTCACTCCAGGTCAATC			
7	bEDA_Ex7_P	GGGGAGTTGGAGGTACTGGT[6FAM]			65
7	bEDA_Ex7_A	[ROX]CGGCACCTACTTCATCTATAGTCAGG[PHOS]			68

Amplicons were analyzed by 1.5% agarose gel electrophoresis at 120 V for 25 minutes and inspected under UV light after staining with ethidium bromide. 5 μL of PCR products were enzymatically cleaned using 1 μL Rapid PCR Cleanup Enzyme Set (NEB, Frankfurt Germany) according to the manufacturers`protocols. Chain termination synthesis was performed using 1 μL of cleaned product, 1 μL of one associated primer, 1 μL BigDye Terminator v3.1, 1 μL 5 x Sequencing buffer (Applied Biosystems, Waltham, MA, USA), and 3 μL water according to the manufacturer´s protocols. Sequencing reactions were separated on an ABI PRISM 3130XL Genetic Analyzer system (Applied Biosystems, Waltham, MA, USA). Sequence data were aligned and analyzed against the NCBI gene reference sequence (GeneID 616179, ARS-UCD1.3) using DNASTAR Lasergene 17 SeqMan Ultra [39–41]. The Effect of identified variants was predicted using the PolyPhen2 v2.2.3 r406 web tool by feeding in the protein sequence, substitution position, and amino acids [42]. The DNA sequence data have deposited with OSF and can be accessed at https://osf.io/rwjh3/.

### FRET genotyping of SNP rs439722471

For SNP rs439722471 probe and anchor were designed using uMelt [43] (Table 1). Oligos were synthesized by Sigma-Aldrich (Taufkirchen, Germany). Fluorescence resonance energy transfer (FRET) melting curve genotyping was performed on 2,016 samples in a LightCycler480 system (Roche, Mannheim, Germany). Each 10 μL reaction mix contained 0.6 U FastStart Taq DNA Polymerase (5 U/μl; Sigma-Aldrich Chemie GmbH, Taufkirchen, Germany), 2 mM dNTP, 4 mM of each primer and probe, PCR reaction buffer (including 15 mM MgCl_2_), and approximately 20 ng of DNA. Cycling conditions were 95°C for 5 minutes, followed by 35 cycles of 95°C for 10 sec, 60°C for 15 sec and 72°C for 15 sec. The final elongation step was 72°C for 5 min. Melting was done using a 498–660 nm detection system initiated with 98°C for 30 sec, 40°C for 1 sec to 80°C with continuous acquisition mode (2/°C), ramp rate 0.29°C/sec, followed by cooling to 40°C for 30 sec. The allele frequency of rs439722471 was determined from a variant calling format (.vcf) file of the X chromosome from Run9 of the 1000 Bull Genomes Project using bcf-tools [44, 45].

### Multispecies alignment of EDA protein sequences

Amino acid sequence alignment of the 391-residue EDA-A1 protein from 13 mammalian species was conducted with the ClustalW algorithm. Protein sequences of bobcats, cattle, cheetahs, Eurasian otters, fishing cats, golden spiny mice, house mice, humans, Jamaican fruit-eating bats, macaque, reed vole, slow loris, and warthog were downloaded from the NCBI protein database [39, 40]. Analysis and visualization were done with DNASTAR Lasergene 17 MegAlign pro [41]. A cartoon model of the bovine EDA-A1 (Q9BEG5) protein was sketched and colorized with UCSF ChimeraX version 1.6.dev202302220544 [46].

## Results

### Clinical examination and histopathology

**Fig 1:**
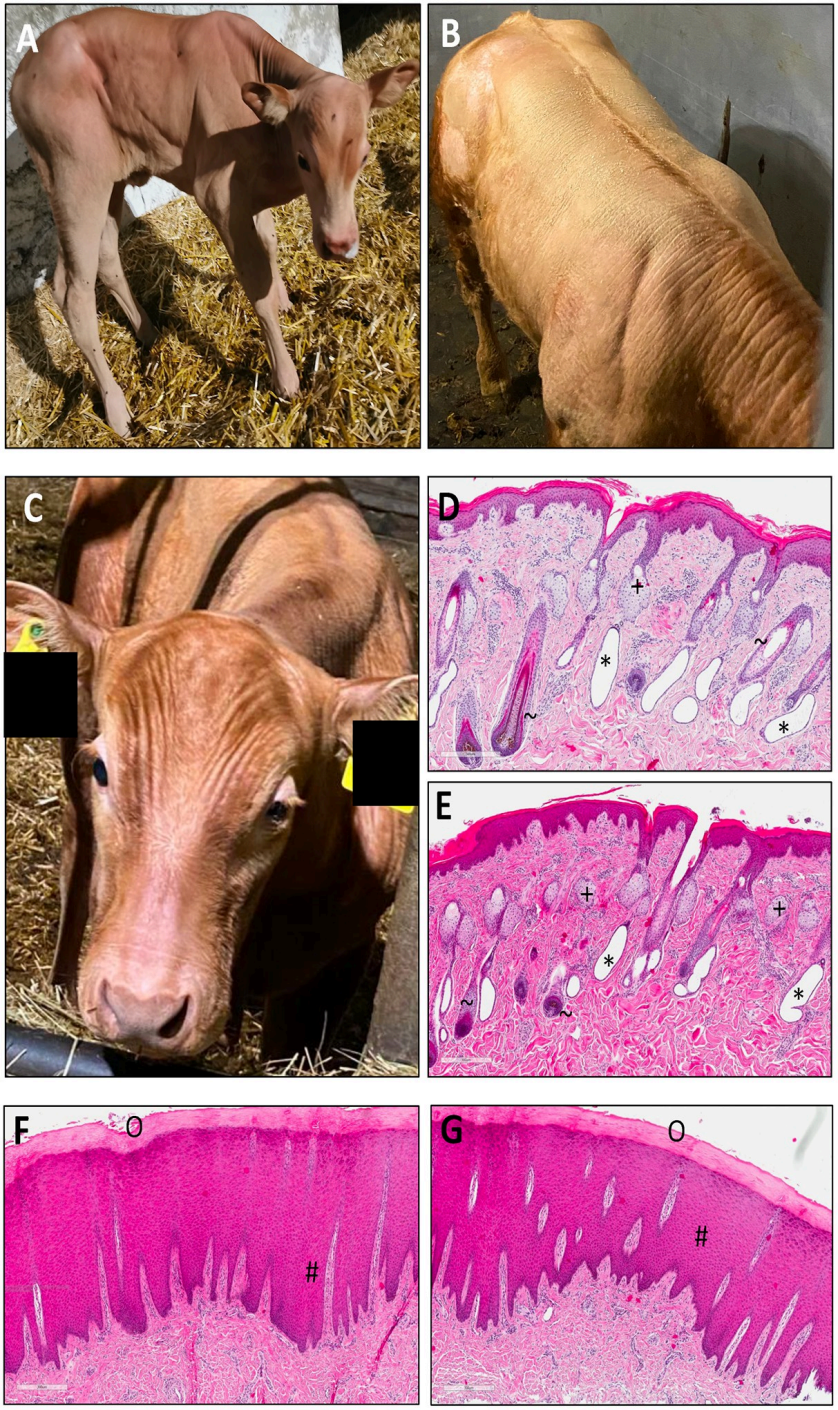
Phenotypical appearance and histopathological examinations of the affected Limousin calf. (A) Photo of the calf at day 1 of age with overall sparse fur. (B-C) Photos of the calf aged six months with overall sparse fur and bald areas around muzzle and eyes. (D-E) Histopathological images of haired area of the affected Calf’s forehead with dilated apocrine glands (*), sebaceous glands (+) and hair follicles (~) after HE staining. (F-G) Histopathological images of bald areas of the forehead showing severe irregular acanthosis (#), hyperkeratosis (O) and lack of any glandular appendices.

**Fig 2:**
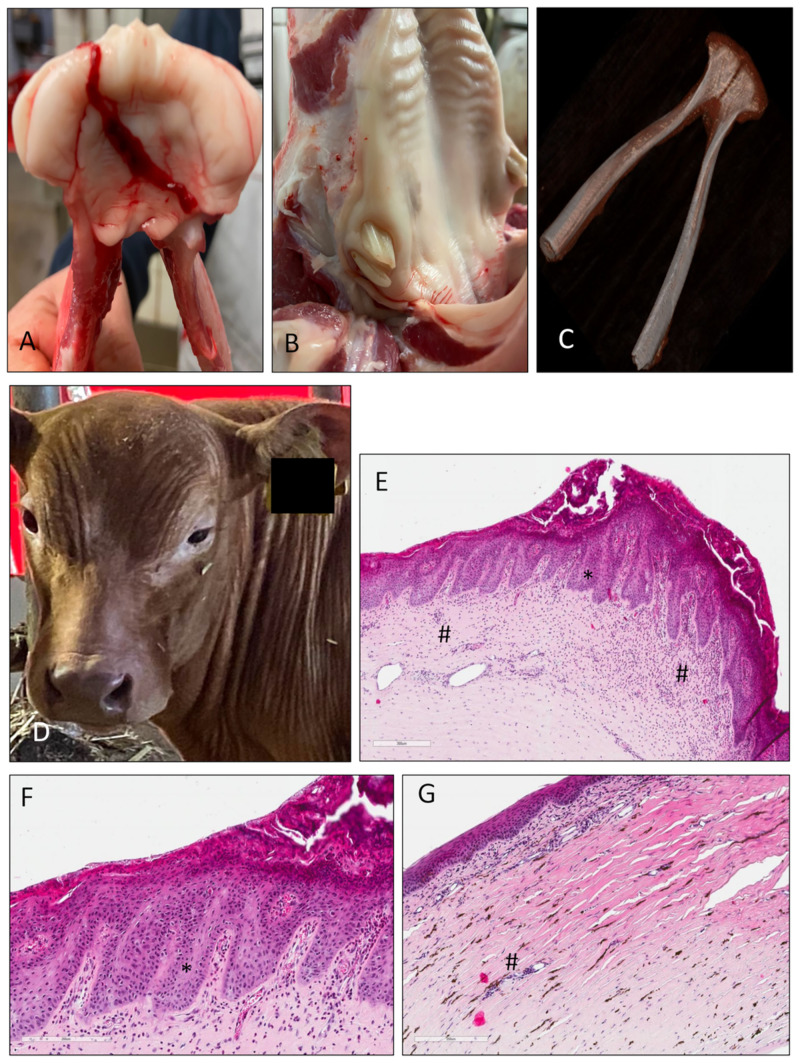
Clinical examinations of the affected Limousin calf. (A-C) Total dental aplasia of teeth except of third molar. M3 is characteristically malformed as described for HED in cattle. (D-G) Keratitis sicca of the left eye showing permanent squint, severe superficial keratitis with corneal hyperplasia indicated by rete ridge formation (*) and invasion of different inflammatory cell populations (#).

Photographs of the affected calf after birth (A) and 5 months later at slaughter (B-C) are shown in Fig 1. The calf was the second case with this clinical presentation within the Limousin cattle family. A previous case that had occurred two years earlier involved an uncle that died at 3 months of age. The affected calf showed overall sparse and dry coat. In particular, the areas around the eyes and muzzle were almost hairless. Fig 1 also shows histopathological images from sparsely hairy (D-E) and bald areas (F-G) of the forehead. The sparsely hairy skin showed mild irregular acanthosis and moderate orthokeratotic lamellar hyperkeratosis, minimal multifocal lymphoplasmacytic inflammatory cell infiltrates and there was evidence of sebaceous glands, hair follicles (including anagen), and dilated apocrine glands. The glabrous skin showed no evidence of sebaceous glands, hair follicles, or apocrine glands with mild perivascular fibrosis and discreet arrector pili muscle. There were also minor multifocal lymphoplasmacytic inflammatory cell infiltrates. Another sign of the affected calf was left-sided blepharospasm, which was visible at birth (Fig 1C). Histopathologic examination of the left eyeball (Fig 2E–2G) revealed focally extensive, eosinophilic, severe superficial keratitis, characterized by hyperplasia of the corneal epithelium (rete ridge formation), infiltration of inflammatory cells (plasma cells, lymphocytes, macrophages, many eosinophils, low numbers of neutrophils). In addition, neovascularization, erosion of the corneal epithelium with epithelial detachment and adherent cell debris (containing foreign material), exocytosis of inflammatory cells, and corneal edema were noted The present clinical and histopathologic findings were suggestive of keratoconjunctivitis sicca. Inspection of the oral cavity revealed no tooth eruption through the gingiva except for a malformed last molar. A computed tomography scan of the mandible showed complete aplasia of all other teeth (Fig 2A–2C). The complete video of the scan can be seen in S1 Fig.

**Fig 3:**
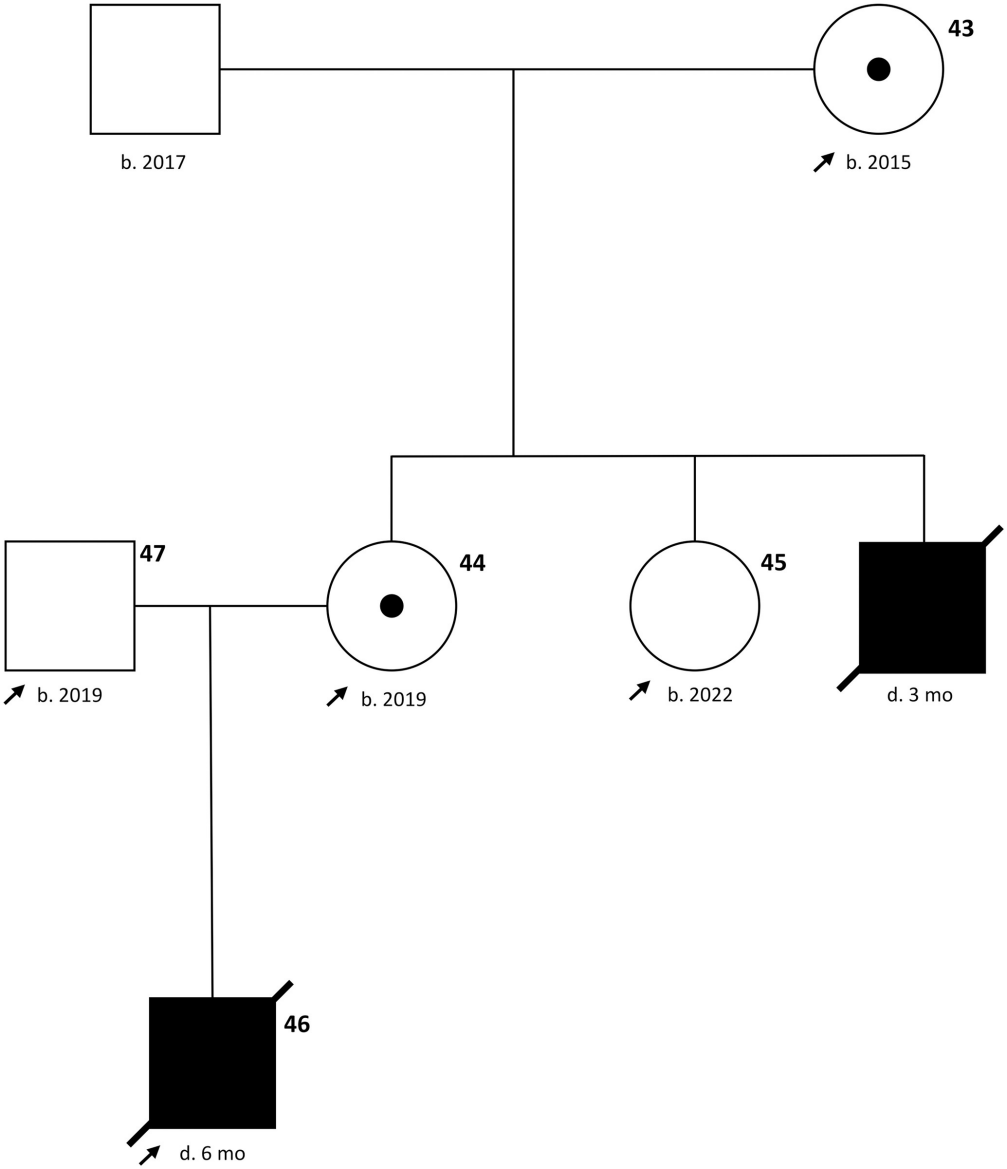
Pedigree of the HED affected Limousin family according to the standardized human pedigree nomenclature [50].

The clinical and histopathological examinations unequivocally allowed the diagnosis of HED. In connection with the pedigree shown in Fig 3, an X-linked inheritance was obvious, because up to now only one other male animal with the same signs had been reported. Therefore, the X-linked *EDA* gene was comparatively sequenced and the sequences were aligned with the reference genome.

### Molecular study

A single missense variant (rs439722471) was identified located in exon 7 at position X:g.80411716T>C (ARS-UCD1.3). The resulting amino acid exchange was predicted to be deleterious according to PolyPhen2 with a score of 0.982 (sensitivity: 0.75; specificity: 0.96) [42]. To test whether this variant was indeed causative of XLHED in the Limousin cattle, a total of 2,013 bovine DNA, including 814 samples from Limousin cattle (40.38%), were genotyped using FRET, and no exchange was detectable in the TNF-like domain, indicating that this region is highly conserved across species (S2 Fig). To further expand the experimental data set, 5,116 multibreed samples from Run9 of the 1000 Bull Genomes Project were also analyzed for the variant. However, the causative C allele could not be detected in these samples either.

**Fig 4:**
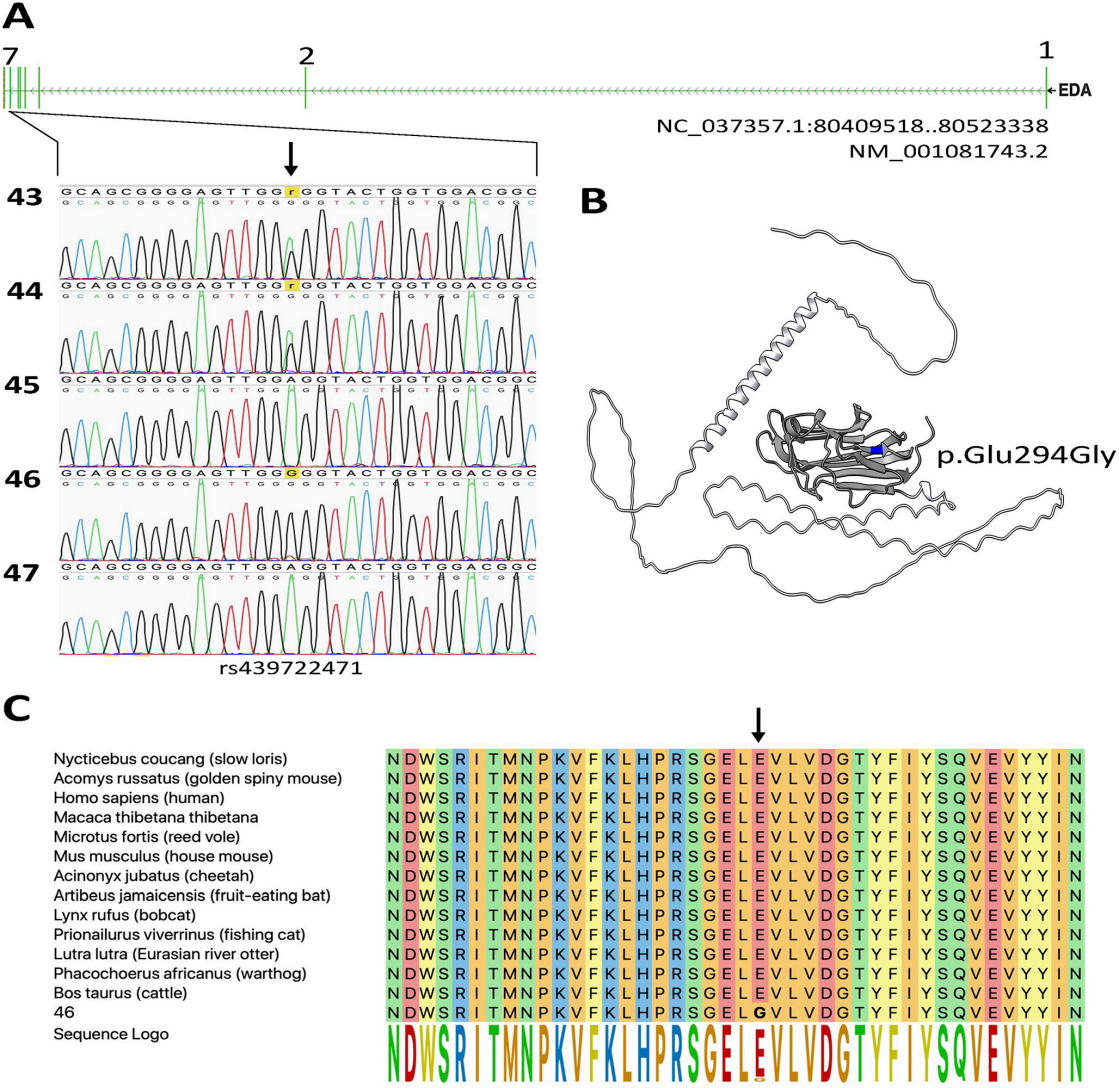
Effect of variant rs439722471. (A) The missense SNP is within exon 7 of EDA transcript NM_001081743.2. Mind the homozygous G-Allele in the calf (46). Numbering 43–47 is equivalent to lab numbers: 43 = grandmother, 44 = mother, 45 = unaffected aunt, 46 = affected Calf, 47 = father. (B) The cartoon model demonstrates in blue the predicted amino acid exchange in the Ectodysplasin A protein from Glutamic acid to Glycine at residue 294. The TNF-like domain is highlighted in grey. (C) Multispecies-42-residues alignment of the EDA protein sequence. Position of the variant rs439722471 (arrow) is within the highly conserved TNF-like domain.

Because the variant was localized in a functionally important region of the protein, we aimed to determine its functional significance by comparative protein analysis in silico. Comparison of the protein sequences of EDA-A1 revealed a total of only 64 exchanges when 13 different mammals were compared, with no exchange detectable in the TNF-like domain. Fig 4 shows a 42-residue window of a multispecies alignment flanking the variant and a cartoon model of EDA-A1.

## Discussion

Hypohidrotic ectodermal dysplasia is accompanied by a characteristic pathology of the coat and teeth. In the present case, these changes were noted in the clinical as well as histopathological and imaging examinations. Similar clinical signs have also been reported in another male individual from this family, although no further investigations were performed. A sign not previously described in cattle HED was observed in the present case in the form of a keratoconjunctivitis sicca. However, this may be due to the fact that cattle with this disorder rarely reach an age at which the disease is expressed. In contrast, in humans and dogs, the occurrence of conjunctivitis sicca associated with HED has been described previously [23]. Because wound healing and also corneal epithelial homeostasis are affected by EDA signaling, the development of conjunctivitis sicca in association with HED is certainly to be expected [47]. These changes are likely exacerbated by decreased or absent tear production due to absent or malformed lacrimal glands. Because lacrimal fluid also has an antimicrobial effect, inflammation and increased risk of infection are also common [48]. In summary, however, the diagnosis of HED can be made without doubt from the overall findings.

Evidence for a sex-linked mode of inheritance came from the available pedigree data and the fact that only males have been affected by the disease to date. Although a dominant form of XLHED due to X inactivation has also been described in humans and cattle in female individuals, a recessive disease was present here because no cows were affected [17, 49]. Based on the overall findings, it was clear that the cause of the disease in the present case could only be linked to the X-linked *EDA* gene. Comparative sequencing of the coding region of the *EDA* gene in the Limousin family revealed a single missense variant. This variant was predicted to result in an amino acid exchange of residue 294 of the EDA-A1 protein from glutamic acid to glycin. The exchange occurs within the TNF-like domain and activation of the ectodysplasin A receptor was predicted to fail because of insufficient ligand-receptor interaction. Of all the bovine HED-causing variants described to date, four are in this region [15–17]. In addition, this variant also likely prevents activation of XEDAR, because EDA-A2 arises from the same gene and also contains the TNF-like domain encoded by the mutant sequence, however, this effect is not observed phenotypically [34, 36]. To prove that the variant was unique and not present in the rest of the population, we genotyped an additional 2,013 bovine DNA samples including 814 Limousin DNA, and screened 5,116 sequence data from the 1000 Bull Genomes Project [44]. The missense variant was not detected in any of the 7,129 samples.

## Conclusions

In conclusion, we were able to confirm the presence of XLHED in a Limousin calf and elucidated a relevant mutation in the EDA gene associated with the defect. Knowledge of this previously undescribed causative variant in cattle now allows the use of a direct genetic test to screen carriers in Limousin breeding.

## Supporting information

S1 Fig Computed tomography scan of the mandible showing complete aplasia of all other teeth. (MOV)

S2 Fig Melting curves of genotypes using FRET assay. Animals carrying the missense variant show a peak at approximately 57°C and the wild type allele is detected with a peak at 65°C. The black line indicates the hemizygous genotype of the affected male calf (46) with only one peak at 57°C. The conductors (mother 44, grandmother 43) are depicted with red colored lines showing two peaks indicating the presence of a heterozygous genotype. The magenta and green colored lines showing a peak at 65°C correspond to the wild type alleles of the homozygous sister (45) and hemizygous father (47). All control samples (blue lines) show only the wild type allele. The cyan colored line corresponds to the non-template control. Numbering according to Fig 3.
(TIF)

## References

[1] Mokhtari S, Mokhtari S, Lotfi A. Christ-siemens-touraine syndrome: a case report and review of the literature. Case Rep Dent. 2012;2012:586418. Epub 2012/12/18. doi: 10.1155/2012/586418 . 23243521 PMC3517823

[2] Srivastava AK, Pispa J, Hartung AJ, Du Y, Ezer S, Jenks T, et al. The Tabby phenotype is caused by mutation in a mouse homologue of the EDA gene that reveals novel mouse and human exons and encodes a protein (ectodysplasin-A) with collagenous domains. Proc Natl Acad Sci U S A. 1997;94(24):13069–74. Epub 1997/12/16. doi: 10.1073/pnas.94.24.13069 . 9371801 PMC24264

[3] Drögemüller C, Distl O, Leeb T. Partial deletion of the bovine ED1 gene causes anhidrotic ectodermal dysplasia in cattle. Genome Res. 2001;11(10):1699–705. Epub 2001/10/10. doi: 10.1101/gr.182501 . 11591646 PMC311120

[4] Drögemüller C, Kuiper H, Peters M, Guionaud S, Distl O, Leeb T. Congenital hypotrichosis with anodontia in cattle: a genetic, clinical and histological analysis. Vet Dermatol. 2002;13(6):307–13. Epub 2002/12/05. doi: 10.1046/j.1365-3164.2002.00313.x . 12464063

[5] Drögemüller C, Peters M, Pohlenz J, Distl O, Leeb T. A single point mutation within the ED1 gene disrupts correct splicing at two different splice sites and leads to anhidrotic ectodermal dysplasia in cattle. J Mol Med (Berl). 2002;80(5):319–23. Epub 2002/05/22. doi: 10.1007/s00109-002-0320-z . 12021844

[6] Drögemüller C, Distl O, Leeb T. X-linked anhidrotic ectodermal dysplasia (ED1) in men, mice, and cattle. Genet Sel Evol. 2003;35 Suppl 1(Suppl 1):S137–45. Epub 2003/08/21. doi: 10.1186/1297-9686-35-S1-S137 . 12927086 PMC3231755

[7] Seeliger F, Drögemüller C, Tegtmeier P, Baumgartner W, Distl O, Leeb T. Ectodysplasin-1 deficiency in a German Holstein bull associated with loss of respiratory mucous glands and chronic rhinotracheitis. J Comp Pathol. 2005;132(4):346–9. Epub 2005/05/17. doi: 10.1016/j.jcpa.2004.11.001 . 15893993

[8] Drögemüller C, Barlund CS, Palmer CW, Leeb T. A novel mutation in the bovine EDA gene causing anhidrotic ectodermal dysplasia (Brief report). Arch Anim Breed. 2006;49(6):615–6. doi: 10.5194/aab-49-615-2006

[9] Barlund CS, Clark EG, Leeb T, Drögemüller C, Palmer CW. Congenital hypotrichosis and partial anodontia in a crossbred beef calf. Can Vet J. 2007;48(6):612–4. Epub 2007/07/10. . 17616058 PMC1876189

[10] Monreal AW, Ferguson BM, Headon DJ, Street SL, Overbeek PA, Zonana J. Mutations in the human homologue of mouse dl cause autosomal recessive and dominant hypohidrotic ectodermal dysplasia. Nat Genet. 1999;22(4):366–9. Epub 1999/08/04. doi: 10.1038/11937 . 10431241

[11] Gargani M, Valentini A, Pariset L. A novel point mutation within the EDA gene causes an exon dropping in mature RNA in Holstein Friesian cattle breed affected by X-linked anhidrotic ectodermal dysplasia. BMC Vet Res. 2011;7:35. Epub 2011/07/12. doi: 10.1186/1746-6148-7-35 . 21740563 PMC3224562

[12] Karlskov-Mortensen P, Cirera S, Nielsen OL, Arnbjerg J, Reibel J, Fredholm M, et al. Exonization of a LINE1 fragment implicated in X-linked hypohidrotic ectodermal dysplasia in cattle. Anim Genet. 2011;42(6):578–84. Epub 2011/11/01. doi: 10.1111/j.1365-2052.2011.02192.x . 22034998

[13] Ogino A, Kohama N, Ishikawa S, Tomita K, Nonaka S, Shimizu K, et al. A novel mutation of the bovine EDA gene associated with anhidrotic ectodermal dysplasia in Holstein cattle. Hereditas. 2011;148(1):46–9. Epub 2011/03/18. doi: 10.1111/j.1601-5223.2010.02202.x . 21410470

[14] Ogino A, Shimizu K, Tanabe Y, Morita M. De novo mutation of the bovine EDA gene associated with anhidrotic ectodermal dysplasia in Japanese Black cattle. Anim Genet. 2012;43(5):646. Epub 2012/04/14. doi: 10.1111/j.1365-2052.2011.02290.x . 22497423

[15] Capuzzello G, Jacinto JGP, Hafliger IM, Chapman GE, Martin SS, Viora L, et al. A large deletion encompassing exon 2 of the ectodysplasin A (EDA) gene in a British blue crossbred calf with hypohidrotic ectodermal dysplasia. Acta Vet Scand. 2022;64(1):23. Epub 2022/09/07. doi: 10.1186/s13028-022-00641-2 . 36068608 PMC9446731

[16] O’Toole D, Hafliger IM, Leuthard F, Schumaker B, Steadman L, Murphy B, et al. X-Linked Hypohidrotic Ectodermal Dysplasia in Crossbred Beef Cattle Due to a Large Deletion in EDA. Animals (Basel). 2021;11(3). Epub 2021/04/04. doi: 10.3390/ani11030657 . 33801223 PMC7999020

[17] Escouflaire C, Rebours E, Charles M, Orellana S, Cano M, Riviere J, et al. Alpha de novo 3.8-Mb inversion affecting the EDA and XIST genes in a heterozygous female calf with generalized hypohidrotic ectodermal dysplasia. BMC Genomics. 2019;20(1):715. Epub 2019/09/20. doi: 10.1186/s12864-019-6087-1 . 31533624 PMC6749632

[18] Casal ML, Scheidt JL, Rhodes JL, Henthorn PS, Werner P. Mutation identification in a canine model of X-linked ectodermal dysplasia. Mamm Genome. 2005;16(7):524–31. Epub 2005/09/10. doi: 10.1007/s00335-004-2463-4 . 16151697 PMC3330241

[19] Bayes M, Hartung AJ, Ezer S, Pispa J, Thesleff I, Srivastava AK, et al. The anhidrotic ectodermal dysplasia gene (EDA) undergoes alternative splicing and encodes ectodysplasin-A with deletion mutations in collagenous repeats. Hum Mol Genet. 1998;7(11):1661–9. Epub 1998/09/16. doi: 10.1093/hmg/7.11.1661 . 9736768

[20] Del-Pozo J, MacIntyre N, Azar A, Headon D, Schneider P, Cheeseman M. Role of ectodysplasin signalling in middle ear and nasal pathology in rat and mouse models of hypohidrotic ectodermal dysplasia. Dis Model Mech. 2019;12(4). Epub 2019/04/28. doi: 10.1242/dmm.037804 . 31028034 PMC6505480

[21] Wright JT, Grange DK, Fete M. Hypohidrotic Ectodermal Dysplasia. In: Adam MP, Mirzaa GM, Pagon RA, Wallace SE, Bean LJH, Gripp KW, Amemiya A, editors. GeneReviews((R)). Seattle (WA)1993. 20301291

[22] Hadji Rasouliha S, Bauer A, Dettwiler M, Welle MM, Leeb T. A frameshift variant in the EDA gene in Dachshunds with X-linked hypohidrotic ectodermal dysplasia. Anim Genet. 2018;49(6):651–4. Epub 2018/10/03. doi: 10.1111/age.12729 . 30276836

[23] Moura E, Rotenberg IS, Pimpao CT. X-Linked Hypohidrotic Ectodermal Dysplasia-General Features and Dental Abnormalities in Affected Dogs Compared With Human Dental Abnormalities. Top Companion Anim Med. 2019;35:11–7. Epub 2019/05/28. doi: 10.1053/j.tcam.2019.03.002 . 31122682

[24] Waluk DP, Zur G, Kaufmann R, Welle MM, Jagannathan V, Drögemüller C, et al. A Splice Defect in the EDA Gene in Dogs with an X-Linked Hypohidrotic Ectodermal Dysplasia (XLHED) Phenotype. G3 (Bethesda). 2016;6(9):2949–54. Epub 2016/07/28. doi: 10.1534/g3.116.033225 . 27449516 PMC5015951

[25] Moura E, Daltro SRT, Sas DM, Engracia Filho JR, Farias MR, Pimpao CT. Genetic analysis of a possible case of canine X-linked ectodermal dysplasia. J Small Anim Pract. 2021;62(12):1127–30. Epub 2021/06/03. doi: 10.1111/jsap.13385 . 34076266

[26] Trzeciak WH, Koczorowski R. Molecular basis of hypohidrotic ectodermal dysplasia: an update. J Appl Genet. 2016;57(1):51–61. Epub 2015/08/22. doi: 10.1007/s13353-015-0307-4 . 26294279 PMC4731439

[27] Hymowitz SG, Compaan DM, Yan M, Wallweber HJ, Dixit VM, Starovasnik MA, et al. The crystal structures of EDA-A1 and EDA-A2: splice variants with distinct receptor specificity. Structure. 2003;11(12):1513–20. Epub 2003/12/06. doi: 10.1016/j.str.2003.11.009 . 14656435

[28] Chen Y, Molloy SS, Thomas L, Gambee J, Bachinger HP, Ferguson B, et al. Mutations within a furin consensus sequence block proteolytic release of ectodysplasin-A and cause X-linked hypohidrotic ectodermal dysplasia. Proc Natl Acad Sci U S A. 2001;98(13):7218–23. Epub 2001/06/21. doi: 10.1073/pnas.131076098 . 11416205 PMC34649

[29] Kwack MH, Kim JC, Kim MK. Ectodysplasin-A2 induces apoptosis in cultured human hair follicle cells and promotes regression of hair follicles in mice. Biochem Biophys Res Commun. 2019;520(2):428–33. Epub 2019/10/15. doi: 10.1016/j.bbrc.2019.10.031 . 31607478

[30] Newton K, French DM, Yan M, Frantz GD, Dixit VM. Myodegeneration in EDA-A2 transgenic mice is prevented by XEDAR deficiency. Mol Cell Biol. 2004;24(4):1608–13. Epub 2004/01/30. doi: 10.1128/MCB.24.4.1608-1613.2004 . 14749376 PMC344191

[31] Yan M, Wang LC, Hymowitz SG, Schilbach S, Lee J, Goddard A, et al. Two-amino acid molecular switch in an epithelial morphogen that regulates binding to two distinct receptors. Science. 2000;290(5491):523–7. Epub 2000/10/20. doi: 10.1126/science.290.5491.523 . 11039935

[32] Bourneuf E, Otz P, Pausch H, Jagannathan V, Michot P, Grohs C, et al. Rapid Discovery of De Novo Deleterious Mutations in Cattle Enhances the Value of Livestock as Model Species. Sci Rep. 2017;7(1):11466. Epub 2017/09/15. doi: 10.1038/s41598-017-11523-3 . 28904385 PMC5597596

[33] Bal E, Baala L, Cluzeau C, El Kerch F, Ouldim K, Hadj-Rabia S, et al. Autosomal dominant anhidrotic ectodermal dysplasias at the EDARADD locus. Hum Mutat. 2007;28(7):703–9. Epub 2007/03/14. doi: 10.1002/humu.20500 . 17354266

[34] Verhelst K, Gardam S, Borghi A, Kreike M, Carpentier I, Beyaert R. XEDAR activates the non-canonical NF-kappaB pathway. Biochem Biophys Res Commun. 2015;465(2):275–80. Epub 2015/08/12. doi: 10.1016/j.bbrc.2015.08.019 . 26260321

[35] Wark AR, Aldea D, Tomizawa RR, Kokalari B, Warder B, Kamberov YG. Ectodysplasin signaling via Xedar is required for mammary gland morphogenesis. J Invest Dermatol. 2023. Epub 2023/02/23. doi: 10.1016/j.jid.2023.02.007 . 36804570 PMC10363239

[36] Wisniewski SA, Trzeciak WH. A new mutation resulting in the truncation of the TRAF6-interacting domain of XEDAR: a possible novel cause of hypohidrotic ectodermal dysplasia. J Med Genet. 2012;49(8):499–501. Epub 2012/08/15. doi: 10.1136/jmedgenet-2012-100877 . 22889853

[37] Adaimy L, Chouery E, Megarbane H, Mroueh S, Delague V, Nicolas E, et al. Mutation in WNT10A is associated with an autosomal recessive ectodermal dysplasia: the odonto-onycho-dermal dysplasia. Am J Hum Genet. 2007;81(4):821–8. Epub 2007/09/12. doi: 10.1086/520064 . 17847007 PMC1973944

[38] Nicholas FW. Online Mendelian Inheritance in Animals (OMIA): a comparative knowledgebase of genetic disorders and other familial traits in non-laboratory animals. Nucleic Acids Res. 2003;31(1):275–7. Epub 2003/01/10. doi: 10.1093/nar/gkg074 . 12520001 PMC165521

[39] O’Leary NA, Wright MW, Brister JR, Ciufo S, Haddad D, McVeigh R, et al. Reference sequence (RefSeq) database at NCBI: current status, taxonomic expansion, and functional annotation. Nucleic Acids Res. 2016;44(D1):D733–45. Epub 2015/11/11. doi: 10.1093/nar/gkv1189 . 26553804 PMC4702849

[40] Sayers EW, Bolton EE, Brister JR, Canese K, Chan J, Comeau DC, et al. Database resources of the national center for biotechnology information. Nucleic Acids Res. 2022;50(D1):D20–D6. doi: 10.1093/nar/gkab1112 . 34850941 PMC8728269

[41] Burland TG. DNASTAR’s Lasergene sequence analysis software. Methods Mol Biol. 2000;132:71–91. doi: 10.1385/1-59259-192-2:71 . 10547832

[42] Adzhubei IA, Schmidt S, Peshkin L, Ramensky VE, Gerasimova A, Bork P, et al. A method and server for predicting damaging missense mutations. Nat Methods. 2010;7(4):248–9. Epub 2010/04/01. doi: 10.1038/nmeth0410-248 . 20354512 PMC2855889

[43] Dwight Z, Palais R, Wittwer CT. uMELT: prediction of high-resolution melting curves and dynamic melting profiles of PCR products in a rich web application. Bioinformatics. 2011;27(7):1019–20. doi: 10.1093/bioinformatics/btr065 21300699

[44] Hayes BJ, Daetwyler HD. 1000 Bull Genomes Project to Map Simple and Complex Genetic Traits in Cattle: Applications and Outcomes. Annu Rev Anim Biosci. 2019;7:89–102. Epub 2018/12/07. doi: 10.1146/annurev-animal-020518-115024 . 30508490

[45] Li H. A statistical framework for SNP calling, mutation discovery, association mapping and population genetical parameter estimation from sequencing data. Bioinformatics. 2011;27(21):2987–93. Epub 2011/09/10. doi: 10.1093/bioinformatics/btr509 . 21903627 PMC3198575

[46] Pettersen EF, Goddard TD, Huang CC, Meng EC, Couch GS, Croll TI, et al. UCSF ChimeraX: Structure visualization for researchers, educators, and developers. Protein Sci. 2021;30(1):70–82. Epub 2020/09/04. doi: 10.1002/pro.3943 . 32881101 PMC7737788

[47] Li S, Zhou J, Bu J, Ning K, Zhang L, Li J, et al. Ectodysplasin A protein promotes corneal epithelial cell proliferation. J Biol Chem. 2017;292(32):13391–401. Epub 2017/06/29. doi: 10.1074/jbc.M117.803809 . 28655773 PMC5555198

[48] Kuony A, Ikkala K, Kalha S, Magalhaes AC, Pirttiniemi A, Michon F. Ectodysplasin-A signaling is a key integrator in the lacrimal gland-cornea feedback loop. Development. 2019;146(14). Epub 2019/06/22. doi: 10.1242/dev.176693 . 31221639

[49] Lexner MO, Bardow A, Juncker I, Jensen LG, Almer L, Kreiborg S, et al. X-linked hypohidrotic ectodermal dysplasia. Genetic and dental findings in 67 Danish patients from 19 families. Clin Genet. 2008;74(3):252–9. Epub 2008/05/31. doi: 10.1111/j.1399-0004.2008.01037.x . 18510547

[50] Bennett RL, French KS, Resta RG, Doyle DL. Standardized human pedigree nomenclature: update and assessment of the recommendations of the National Society of Genetic Counselors. J Genet Couns. 2008;17(5):424–33. Epub 2008/09/17. doi: 10.1007/s10897-008-9169-9 . 18792771

