# Supplementary figures and images for: A missense mutation in the highly conserved TNF-like domain of Ectodysplasin A is the candidate causative variant for X-linked hypohidrotic ectodermal dysplasia in Limousin cattle: Clinical, histological, and molecular analyses
Source: PLoS One. 2024 Jan 22;19(1):e0291411. doi: 10.1371/journal.pone.0291411 (PMC10802946; doi:10.1371/journal.pone.0291411)

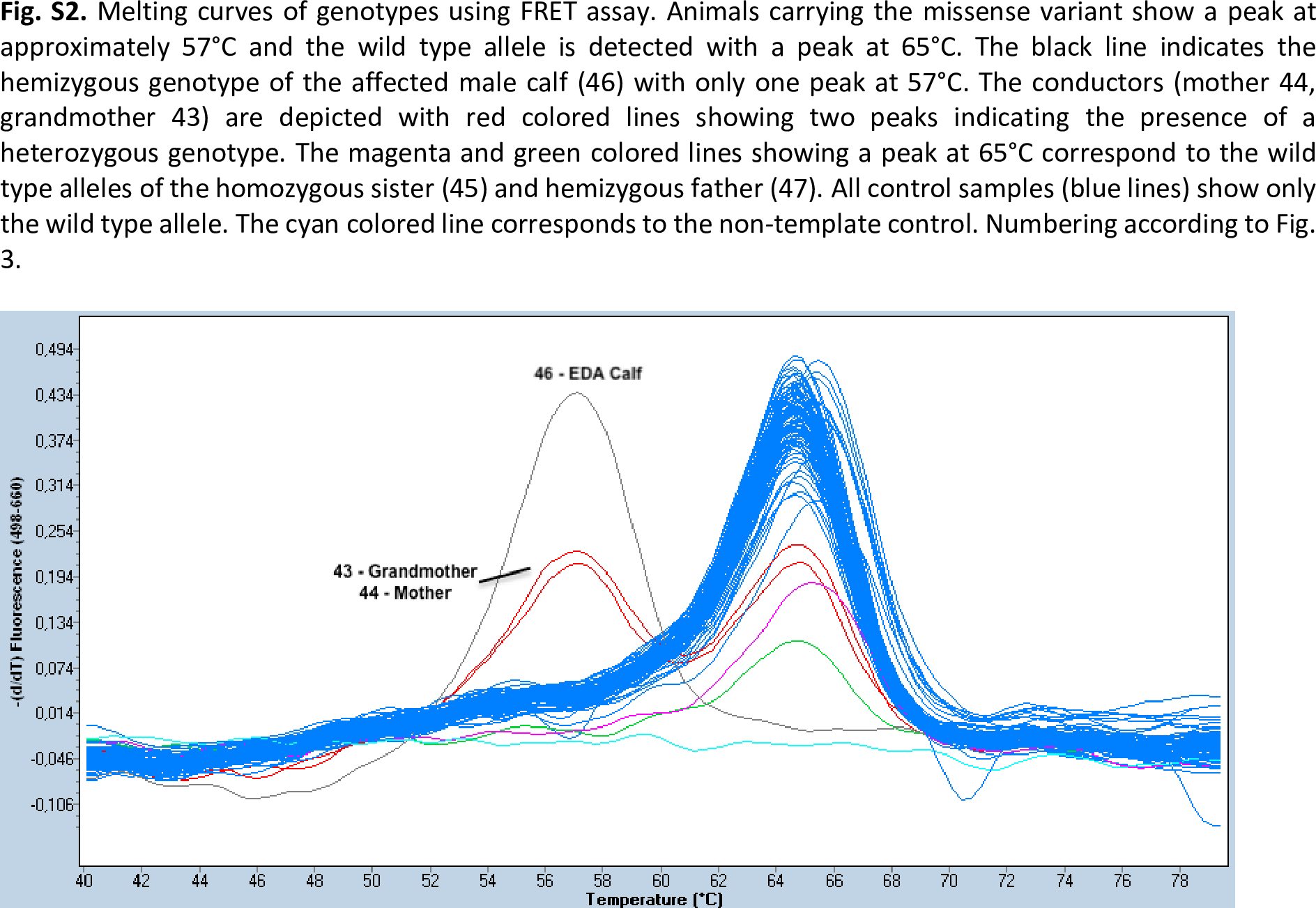

Supplement: S2 Fig — Animals carrying the missense variant show a peak at approximately 57°C and the wild type allele is detected with a peak at 65°C. The black line indicates the hemizygous genotype of the affected male calf (46) with only one peak at 57°C. The conductors (mother 44, grandmother 43) are depicted with red colored lines showing two peaks indicating the presence of a heterozygous genotype. The magenta and green colored lines showing a peak at 65°C correspond to the wild type alleles of the homozygous sister (45) and hemizygous father (47). All control samples (blue lines) show only the wild type allele. The cyan colored line corresponds to the non-template control. Numbering according to Fig 3. (TIF) [file pone.0291411.s002.tif]
